# Chronic Kidney Disease Prevalence in Patients with Colorectal Cancer Undergoing Surgery

**DOI:** 10.3390/diagnostics12092137

**Published:** 2022-09-02

**Authors:** Leszek Kozlowski, Katarzyna Bielawska, Alena Zhymaila, Jolanta Malyszko

**Affiliations:** 1 Oncological Surgery Department with Specialized Cancer Treatment Units, Maria Sklodowska-Curie Bialystok Oncology Centre, 15-027 Białystok, Poland; 2 Department of Nephrology, Dialysis & Internal Diseases, The Medical University of Warsaw, 02-091 Warszawa, Poland

**Keywords:** colorectal cancer, chronic kidney disease, radiochemotherapy, radiotherapy

## Abstract

Colorectal cancer (CRC) is a common and mortal disease. Chronic kidney disease (CKD) is the relatively common comorbidity among cancer patients affecting the available therapy and outcomes. However, data on prevalence of CKD in patients with CRC undergoing surgery is limited. The aim of the study was to evaluate the prevalence of CKD in a cohort of 560 consecutive patients with CRC undergoing surgical treatment with curative intent. Neoadjuvant therapy in a form of radiotherapy or radiochemotherapy was administered before the surgery in 67 patients and in 86 patients, respectively. Results: CKD was reported in 10%, diabetes in 25%, and hypertension in 60%, while anemia was reported in 47%. The patients with CKD were more likely to be older and anemic with higher serum CRP, which reflects a general inflammatory state. Relative to patients without this therapy, patients undergoing neoadjuvant radiochemotherapy were older, had significantly lower eGFR and albumin, and higher creatinine, aspartate aminotransferase and INR, before the surgery. All CKD patients, except two, were older than 65 years of age. Conclusions: In order to ensure the best possible outcomes, CKD should be diagnosed and treated appropriately in oncology patients to prevent complications, so they may continue their therapy with the least interruption or discontinuation of treatment.

## 1. Introduction

Colorectal cancer (CRC) is a common and mortal disease. Annually, more than 1 million people develop CRC worldwide [1]. In addition, the mortality rate related to CRC is almost 33% in developed countries [1], and it is the second leading cause of cancer-related death in the US [2]. Chronic kidney disease (CKD) is the relatively common comorbidity among cancer patients affecting the available therapy and outcomes [3]. On the other hand, CKD has been shown to be associated with the increased risk of CRC [4]. CKD is prevalent worldwide and has high incidences of 13.1% in the US and 11.9% in Poland among individuals older than 20 years of age [5,6]. As reported by Oh et al. [7] females with both CKD and DM had significantly higher risk of CRC, compared with having CKD or DM alone. This significant difference was not observed in males [7]. Nozawa et al. [8] investigated 1127 consecutive patients with stages 0 to III primary colorectal cancer who underwent curative resection from January 2001 to December 2010. They found that 245 (21.8%) patients had CKD stages III to V. They reported no differences in the frequency of adjuvant chemotherapy and recurrence-free survival among different chronic kidney disease stages. Surgery with curative intent plays a crucial role in the treatment of colorectal cancer (CRC). In our pilot study on 100 consecutive patients with CRC undergoing primary surgery with curative intent within 1 year in the Department of Oncological Surgery (with no prior neoadjuvant therapy), prevalence of CKD was 15% [9]. Huang et al. [10] studied 429 patients who underwent curative resection for stages I–III colorectal adenocarcinoma and evaluated the impact of CKD on carcinoembryonic antigen prognostic accuracy in colorectal cancer. In their group, CKD was present preoperatively in 115 patients (27%).

Taking all of these available studies into consideration, and the fact that data on incidence and prevalence of CKD in patients with CRC undergoing surgery is limited, the aim of our study was to assess the prevalence of CKD among colorectal cancer surgery patients and its relation to the neoadjuvant therapy or no prior therapy.

## 2. Patients and Methods

A total of 560 consecutive patients from the regional oncology center, undergoing CRC surgery with curative intent in a period of two years, were included in the retrospective, observational cohort study. Electronic medical records of the included patients were retrospectively reviewed. We collected demographic and clinical data at the time of admission for surgery, such as age, sex, body mass index (BMI), comorbidities (hypertension, diabetes, and cardiovascular disease), and types of surgery (rectal resection, colon resection); as well as laboratory data, such as hemoglobin, albumin, total protein, C-Reactive Protein, urea, creatinine levels, and the estimated glomerular filtration rate (eGFR). Blood pressure was measured with standard protocols. Hypertension was defined as either a blood pressure of at least 140/90 mm Hg or hypotensive medication use. According to the criteria established by the Kidney Disease Outcome Quality Initiative and Kidney Disease Improving Global Outcome guideline, CKD was defined as kidney damage or eGFR < 60 mL/min/1.73 m^2^ lasting for three months or more, irrespective of the cause [11], with eGFR estimated using CKD-EPI formula [12]. Anemia was diagnosed according to World Health Organization (WHO) criteria, i.e., respectively in men hemoglobin < 13 g/dL and women < 12 g/dL. [13]. Preoperative therapy was also investigated. Neoadjuvant radiochemotherapy was administered to 86 patients (50 Gy in 25 fractions of 2 Gy, followed by chemotherapy according to CAPOX protocol, i.e., capecitabine plus oxaliplatin) [14], and 67 patients underwent neoadjuvant radiotherapy (5 × 5 Gy up to total dose of 25 Gy). No neoadjuvant therapy was administered to 407 patients prior the surgery. Adenocarcinoma was diagnosed in all treated patients by endoscopy before qualifying for surgery. Patients’ clinical staging was determined on the basis of the criteria of the International Union Against Cancer, VIII edition. The study was approved by the appropriate ethics review board and is compliant with the Declaration of Helsinki. As only retrospective medical data were analyzed, informed consent was waived and positive opinion of the Ethics Committee was obtained.

Data given were analyzed using Statistica 13.1 computer software (Tulsa, OK, USA). Normality of variable distribution was tested using Shapiro–Wilk W-test. Student’s *t*-test was used in statistical analysis to compare differences between groups with *p* < 0.05 considered statistically significant, when appropriate.

## 3. Results

In Table 1, the clinical and biochemical characteristics of the studied population is given. Table 2 presents data in relation to presence of CKD. All CKD patients, except two, were older than 65 years of age. The patients with CKD were more likely to be older and anemic, with lower albumin, longer prothrombin time, lower INR, and higher serum urea and CRP, which reflects a general inflammatory state. When compared to patients without this therapy, patients undergoing neoadjuvant radiochemotherapy were older, had significantly lower eGFR and albumin, and higher creatinine, aspartate aminotransferase and INR, before the surgery (Table 3). Patients undergoing neoadjuvant radiotherapy did not differ in eGFR with patients without neoadjuvant treatment. In our study, 4.7% of patients treated with radiochemotherapy had CKD, while 3% of patients undergoing radiotherapy also had CKD as a comorbidity. In patients treated with neoadjuvant radiochemotherapy, lower eGFR, lower albumin, higher creatinine, higher aspartate aminotransferase, and INR was found before the surgery, when compared to patients without this therapy (Table 3). Patients undergoing neoadjuvant radiotherapy did not differ in eGFR with patients without neoadjuvant treatment. Anemic patients were significantly older and more malnourished (as reflected by lower serum albumin and total protein, and higher activity of aminotransferases and GGTP), and more inflamed (as reflected by higher CRP and leukocyte counts) (Table 4). In addition, they had lower MCH, MCV, and MPV when compared to non-anemic patients. Among the anemia patients, 75% had CKD as a comorbidity.

## 4. Discussion

We found that CKD prevalence in patients with CRC undergoing surgery was 10%. There are not published data on the prevalence of CKD in CRC patients in relation to general population. Moreover, epidemiology of CKD in general population in Poland came from the study performed over 10 years ago in North Poland [15]. Albuminuria was detected in 15.6% of the investigated population (out of 9700 invited subjects, 2471 individuals participated in the PolNef study) using the dipstick test, and thereafter confirmed in 11.9% by the turbidimetric method [15]. We used only eGFR to assess the prevalence of CKD, as albuminuria was not available. In addition, albuminuria is not examined in everyday clinical practice, except by diabetologist in patients with type 1 diabetes as a screening. In addition, patients with neoadjuvant radiochemotherapy had worse kidney function before the surgery (as reflected by a higher serum creatinine and lower eGFR when compared to patients without neoadjuvant therapy or neoadjuvant radiotherapy). All of the patients received CAPOX scheme, as per protocol based on ESMO 2020 guidelines [14]. Platin derivatives, including oxaliplatin, are nephrotoxic [16]. Oxaliplatin is a third-generation platinum-based chemotherapy and is the only agent among this newer group of platinum-based agents to have widespread use in routine cancer therapy, including CRC. Similarly, to other platinum-based anticancer drugs, oxaliplatin exerts its therapeutic effect via blockade of DNA production, forming cross-links in DNA, resulting in cell cycle arrest and apoptosis [17]. Despite being less nephrotoxic than both cisplatin and carboplatin, all platinum-based protocols may cause proximal tubular cell injury [18,19] as well as other renal pathologies, such as acute tubulointerstitial nephritis [20].

In the recent review by Jagiela et al. [21], the authors also stated that data on the nephrotoxicity of oxaliplatin can be found in the literature and cited two case reports [22,23]. In the study by Rödel et al. [24], among 104 patients who received neoadjuvant chemotherapy, 17 patients (16%) experienced an increase in creatinine levels, including 16 cases (15%) which were classified as grade 1/2 and 1 case (1%) classified as grade 3. They also reported that 13 patients (18%) experienced elevated creatinine levels, while 12 patients (16%) had grade 1/2 proteinuria. Prevalence of CKD was not provided. The use of radiotherapy (sometimes in combination with chemotherapy) in the treatment of some cancers, lymphoma and sarcoma may also lead to impairment of renal function [25]. Mechanistically, radiotherapy-induced oxidative damage of DNA results in delayed proteinuria, hypertension, and the impaired ability to concentrate urine [25]. Therefore, using neoadjuvant radiochemotherapy with platinum derivatives may results in impaired kidney function prior to surgery.

Surgery plays a crucial role in the treatment of CRC. As the median age is above 65 years at the time of CRC diagnosis in many studies, surgery is often performed in elderly patients with multimorbidities [26,27,28,29]. It may explain the wide range of complications, including CKD, acute kidney injury or acute kidney disease [30,31]. It should be taken into account the heterogeneity of the available studies in regard to the procedures, urgency of surgery, neoadjuvant treatment concomitant medication, and imaging studies. Launay-Vacher et al. [32] reported that only 27.2% of patients with colorectal cancer (data from the IRMA-1 study) had a GFR ≥ 90 mL/min/1.73 m^2^. However, at the figure presented in the paper, prevalence of CKD in CRC is more than 10% [32]. Velciov et al. [33] reported that in patients with colorectal cancer in county hospital in Western Romania, eGFR by CKD-EPI formula of <60 mL/min/1.73 m^2^ was found in 31/180 patients (17.22% of the cases). In our much larger population, studied CKD was diagnosed in 10%. Teng et al. [34] using the Taiwanese National Health Insurance Research Database retrieved and retrospectively reviewed the records of patients aged ≥55 years who were diagnosed with colon cancer between 2000 and 2005. They found that CKD was present in 4.6% of patients with iron deficiency anemia (n = 1260) vs. 2.2% of patients without iron deficiency anemia (n = 15912). Prevalence of CKD in total cohort was 2.4%, diabetes in 20.9% and hypertension in 45.4% (lower than in our study, despite similar mean age). Prevalence of anemia was not provided. Very recently, Cheng et al. [35] retrieved data of 5135 patients from Chang Gung Research Database (CGRD) in Taiwan undergoing curative surgery between January 2004 to April 2018. Prevalence of CKD in total cohort was 4.0%, diabetes in 22.3% and hypertension in 44% (similar to a previous report from Taiwan [28] and lower than in our study). Batra et al. [36] identified 7841 patients diagnosed with stage II/III colon cancer between 2004 and 2015 from a large province in Canada. They underline that patients age > 75 years, with anemia, comorbid conditions (heart disease, uncontrolled diabetes, kidney disease, and liver disease), and a history of malignancy or immunosuppression (58.6%) were considered ineligible for adjuvant chemotherapy trials in stage II and III colon cancer. In this group, renal dysfunction was present in 26.9% and was the one second most common, after age (36.2%) and before cardiac disease (17.4%) as reason for ineligibility.

Chronic kidney disease is a risk factor of mortality and cardiovascular disease in the general population [37]. It is also reported as being common among cancer patients [3,4,38,39]. Moreover, prior CKD may adversely affect outcomes in patients with CRC [40]. In addition, preexisting heart and kidney impairment may increase the risk of cardiotoxicity of the oncologic treatment, including 5-fluorouracil and capecitabine [41,42]. In our study, patients were treated with capecitabine with oxaliplatin. Ho et al. [43], in the retrospective population-based cohort study of patients with colorectal cancer selected from the National Health Insurance Research Database (NHIRD), assessed the cardiotoxicity after receiving anticancer treatment. In their cohort they found that the prevalence of CKD was 4.6% in the group treated with chemotherapy and 4.7% in the group receiving combined treatment with chemotherapy and targeted therapy. In the chemotherapy group, 65.5% also received radiotherapy and 53.5% received radiotherapy in combination group. In our study, 4.7% of patients treated with radiochemotherapy had CKD, 3% of patients underwent radiotherapy. As stressed by Ho et al. [43], patients with CRC over ≥60 years with a history of hypertension and CKD were at a higher risk of developing cardiotoxicity when treated with anticancer drugs. It should also be pointed out that worsening or de novo systemic hypertension can be found with numerous chemotherapeutics, with antiangiogenic drugs in particular [44]. Moreover, the possible role of complex drug–drug interactions on the risk of cardio- and/or nephrotoxicity could not be excluded [45]. It should also be stressed that alternative medicines, self-paid medications or herbal medicine might also affect heart or kidney function and are not registered in the hospital files.

We also found that almost 50% of patients were anemic. They were more inflamed and more malnourished, with elevated liver enzymes (aminotransferases and GGTP) as well as with MCH and lower MCV indicating iron deficiency (could both be absolute due to probable microbleeding and/or functional due to inflammation). Lower MPV which might reflect lower platelet production by the bone marrow due to chemotherapy. We used WHO criteria to diagnose anemia [13]. These criteria were not created to be used as a gold standard for the anemia diagnosis, they were a part of international nutrition WHO studies [46]. WHO’s definition of anemia and established reference point for anemia are appropriate to apply across populations [47], and currently are widely used in clinical practice.

Anemia is a non-specific sign of possible cancer, and its present in 17% of persons over 65 years of age [48]. It was reported that iron deficiency anemia was a well-established marker of increased risk of gastric and colorectal cancers ranging up to 10% [49,50,51,52]. Diagnosis of anemia prompts further investigation in certain age groups [53]. In addition, anemia, predominantly mild (75%) is found in 39% of subjects diagnosed with cancer [54]. It should also be stressed that anemia is a negative prognostic factor for survival of several types of cancer [55]. However, new data on anemia prevalence in CRC in patients undergoing curative surgery is limited. Kim et al. [56] assessed the relation between cancer and anemia in 502 patients (52 males, 450 females). They found that cancer prevalence among anemia patients was 5.57% (25.0%, men; 3.3%, women). In addition, the most frequently diagnosed cancer was colorectal cancer (22.5%), followed by advanced gastric cancer (16.1%), breast cancer (9.6%), myelodysplastic syndrome (9.6%), cervical cancer (6.4%), renal-cell carcinoma (6.4%), and thyroid cancer (6.4%). Most recently, Altintas et al. [57] studied retrospectively 352 patients who underwent elective curative surgery for CRC between January 2015 and December 2020. They found that 50.3% were postoperatively diagnosed with anemia. In observational population-based cohort study, using individually linked electronic data from laboratory information systems and nationwide healthcare registries in Denmark, 48925 persons aged 40–90 years without a prior history of cancer and with new-onset anemia (no anemia during the previous 15 months) detected in general practice in 2014–2018 were included [58]. Number of cancer cases was 3285 (6.7%) with gastrointestinal cancer being the most frequent cancer type in both men (2.7%) and women (2.2%).

### Limitations of the Study

Our study has several limitations. First, this is a single center, retrospective observational study. Second, only CRC patients undergoing surgery with curative intent were assessed. Only available medical records at the hospital were analyzed. On the other hand, single center study, with one established protocol, surgery techniques, management and data source could be a benefit. Having the population in our study consist of all of the elective cases in a specific region without referrals from other regions (except some urgent admission) is a clear strength in this regard.

## 5. Conclusions

CKD is found in approximately 10% of patients undergoing CRC surgery, and neoadjuvant radiochemotherapy is associated with impaired kidney function. Increase in serum creatinine during chemotherapy may lead to a reduction in the dose, or even to temporary or permanent discontinuation of the drug, and worsen outcomes. Proper hydration is a commonly used and recommended method to prevent nephrotoxicity. However, no specific treatment is available to cope with nephrotoxicity of radiotherapy. Therefore, physicians should carefully evaluate patients for the presence of comorbidities, such as CKD and hypertension, as they may impact outcomes and adjuvant therapy with possible cardiac and renal toxicities of anticancer therapy.

## Figures and Tables

**Table 1:** Clinical and biochemical data of the studied patients with colorectal cancer undergoing surgery.

	n = 560
Age (years)	67.31 ± 10.10
Diabetes (%)	25
Chronic kidney disease	10
Hypertension	60
Albumin (g/dL)	4.30 ± 0.46
Sodium (mmol/L)	139.71 ± 2.58
Potassium (mmol/L)	4.52 ± 0.40
Hematocrit (%)	35.54 ± 5.16
Hemoglobin (g/dL)	12.34 ± 1.96
Erythrocyte count (×10^12^/L)	4.25 ± 0.49
MCV (fl)	83.19 ± 10.67
MCH (pg)	28.57 ± 3.62
MPV (µm^3^)	7.25 ± 1.29
Leukocyte count (109/L)	6.79 ± 2.23
Platelet count (109/L)	269.54 ± 80.97
Creatinine (mg/dL)	0.87 ± 0.26
Urea (mg/dL)	35.58 ± 12.82
eGFR by CKD-EPI (ml/min/1.72 m^2^)	88.33 ± 21.50
PT (s)	12.44 ± 0.96
INR	1.05 ± 0.09
Total protein (g/dL)	6.83 ± 0.54
CRP (mg/L)	3.8 (1.55; 18.95)
Glucose (mg/dL)	110.82 ± 29.08
ALT (U/L)	14 (10; 20)
AST (U/L)	16 (13; 19)
GGTP (U/L)	20 (15; 28.5)

Data given are means ± SD, or medians with interquartile ranges. ALT is defined as alanine aminotransferase, AST is defined as aspartate aminotransferase, GGTP is defined as gamma-glutamyl transpeptidase, MCV is defined as mean corpuscular volume, MCH is defined as mean hemoglobin content, RDW is defined as red cell distribution width, PT is defined as prothrombin time, INR is defined as international normalized ratio, eGFR is defined as estimated glomerular filtration rate, CKD-EPI is defined as chronic kidney disease-epidemiological collaboration, and CRP is defined as C-Reactive protein.

**Table 2:** Clinical and biochemical data of colorectal cancer patients with and without chronic kidney disease CKD.

	No CKD n = 506	CKD n = 54
Age (years)	66.34 ± 9.98	73.43 ± 8.97 ***
Albumin (g/dL)	4.54 ± 0.46	4.23 ± 0.55 **
Sodium (mmol/L)	139.73 ± 2.50	139.50 ± 3.44
Potassium (mmol/L)	4.51 ± 0.38	4.61 ± 0.60
Hematocrit (%)	35.66 ± 5.11	34.24 ± 5.78
Hemoglobin (g/dL)	12.43 ± 1.93	11.35 ± 1.3 3 *
Erythrocyte count (×10^12^/L)	4.28 ± 0.46	3.94 ± 0.67 *
MCV (fl)	82.81 ± 10.84	87.13 ± 7.83
MCH (pg)	28.48 ± 3.61	29.46 ± 3.71
MPV (µm^3^)	7.23 ± 1.28	7.18 ± 0.66
Leukocyte count (10^9^/L)	6.87 ± 2.28	5.89 ± 1.25
Platelet count (10^9^/L)	271.16 ± 82.61	252.13 ± 60.11
Creatinine (mg/dL)	86.63 ± 15.80	102.54 ± 32.71 ***
Urea (mg/dL)	34.00 ± 10.72	51.33 ± 19.88 *
eGFR by CKD-EPI (ml/min/1.72 m^2^)	87.95 ± 18.74	66.32 ± 21.98 ***
PT (s)	12.44 ± 0.96	11.83 ± 0.77 *
INR	1.06 ± 0.09	1.00 ± 0.07 *
Total protein (g/dL)	6.83 ± 0.55	6.80 ± 0.48
CRP (mg/L)	3.2(1.4; 7.2)	12.4 (2.75; 38.4) ***
Glucose (mg/dL)	110.55 ± 26.25	113.71 ±51.85
ALT (U/L)	15 (10; 21)	14 (8; 20)
AST (U/L)	16 (12; 18)	18 (14; 22)
GGTP (U/L)	19 (14; 27)	20 (16; 29)

Data given are means ± SD, or medians with interquartile ranges. ALT is defined as alanine aminotransferase, AST is defined as aspartate aminotransferase, GGTP is defined as gamma-glutamyl transpeptidase, MCV is defined as mean corpuscular volume, MCH is defined as mean hemoglobin content, MPV is defined as mean platelet volume, PT is defined as prothrombin time, INR is defined as international normalized ratio, eGFR is defined as estimated glomerular filtration rate, CKD-EPI is defined as chronic kidney disease-epidemiological collaboration, and CRP is defined as C-Reactive protein. * *p* < 0.05, ** *p* < 0.01, *** *p* < 0.001.

**Table 3:** Clinical and biochemical data of colorectal cancer patients in regard to neoadjuvant therapy.

	No RTH + CHT n = 474	RTH + CHT n = 86
Age (years)	65.01 ± 8.00	67.38 ± 10.41 **
Albumin (g/dL)	4.78 ± 0.11	4.28 ± 0.48 **
Sodium (mmol/L)	139.89 ± 2.67	139.68 ± 2.57
Potassium (mmol/L)	4.42 ± 0.44	4.54 ± 0.39
Hematocrit (%)	36.64 ± 4.61	35.31 ± 5.26
Hemoglobin (g/dL)	12.79 ± 1.46	12.26 ± 2.02
Erythrocyte count (×10^12^/L)	4.28 ± 0.51	4.11 ± 0.48
MCV (fl)	85.89 ± 6.65	82.60 ± 8.89
MCH (pg)	31.03 ± 1.54	28.04 ± 3.72 ***
MCHC (g/dL)	33.65 ± 2.32	31.68 ± 2.76
MPV (µm^3^)	6.89 ± 1.08	7.30 ± 1.27
Leukocyte count (10^9^/L)	6.20 ± 1.71	6.91 ± 2.31
Platelet count (10^9^/L)	267.38 ± 60.65	270.00 ± 84.86
Creatinine (mg/dL)	0.83 ± 0.27	0.98 ± 0.14
Total protein (g/dL)	6.83 ± 0.47	6.79 ± 0.56
eGFR by CKD-EPI (mL/min/1.72 m^2^)	92.58 ± 18.25	87.61 ± 21.95 *
PT (s)	12.14 ± 0.68	12.43 ± 1.01
INR	1.02 ± 0.07	1.06 ± 0.09 *
urea (g/dL)	35.87 ± 13.56	42.76 ± 12.87 *
CRP (mg/L)	1.75 (1.0; 4.9)	4.6 (1.8; 22.7) *
Glucose (mg/dL)	108.59 ± 33.23	121.17 ± 27.73 *
ALT (U/L)	15 (9; 20)	17 (12; 22)
AST (U/L)	14 (11; 17)	16 (12; 20)
GGTP (U/L)	22 (12; 26)	25 (16; 31)

Data given are means ± SD, or medians with interquartile ranges. ALT is defined as alanine aminotransferase, AST is defined as aspartate aminotransferase, GGTP is defined as gamma-glutamyl transpeptidase, MCV is defined as mean corpuscular volume, MCH is defined as mean hemoglobin content, MCHC is defined as mean corpuscular hemoglobin concentration, MPV is defined as mean platelet volume, PT is defined as prothrombin time, INR is defined as international normalized ratio, eGFR is defined as estimated glomerular filtration rate, CKD-EPI is defined as chronic kidney disease-epidemiological collaboration, CRP is defined as C-Reactive protein, RTH is defined as radiotherapy, and CHT is defined as chemotherapy. * p < 0.05, ** *p* < 0.01, *** *p* < 0.001.

**Table 4:** Clinical and biochemical data of colorectal cancer patients in regard to presence or absence of anemia.

	No Anemia n = 298	Anemia n = 262
Age (years)	65.80 ± 9.28	68.33 ± 11.59 *
Albumin (g/dL)	4.43 ± 0.39	4.205 ± 0.49 ***
Sodium (mmol/L)	140.23 ± 2.33	138.85 ± 2.77 ***
Potassium (mmol/L)	4.51 ± 0.41	4.53 ± 0.40
Chloride (mmol/L)	103.28 ± 2.76	103.11 ± 3.37
Hematocrit (%)	38.99 ± 3.61	30.59 ± 3.35 ***
Hemoglobin (g/dL)	13.84 ± 0.96	10.25 ± 1.29 ***
Erythrocyte count (×10^12^/L)	4.47 ± 0.38	4.02 ± 0.48 ***
MCV (fl)	86.30 ± 10.02	77.85 ± 8.88 *
MCH (pg)	30.28 ± 1.69	25.65 ± 4.14 **
MCHC (g/dL)	34.78 ± 1.78	32.65 ± 2.27
MPV (µm^3^)	6.89 ± 1.08	7.30 ± 1.27
Leukocyte count (10^9^/L)	6.62 ± 2.00	7.42 ± 2.67 *
Platelet count (10^9^/L)	246.11 ± 60.01	309.51 ± 75.72 *
Creatinine (mg/dL)	0.80 ± 0.23	0.86 ± 0.27
Total protein (g/dL)	6.93 ± 0.51	6.64 ± 0.35 ***
eGFR by CKD-EPI (mL/min/1.72 m^2^)	92.58 ± 18.25	87.61 ± 21.95 *
PT (s)	12.16 ± 0.79	12.79 ± 1.11
INR	1.03 ± 0.07	1.10 ± 0.10
urea (g/dL)	34.06 ± 11.26	37.42 ± 14.28
CRP (mg/L)	2.3 (1.4; 6.0)	14.3 (3.2; 42.6)
Glucose (mg/dL)	110.76 ± 29.40	110.92 ± 28.74
ALT (U/L)	14 (12; 18)	16 (13; 20) *
AST (U/L)	11 (9; 15)	15 (11; 21)
GGTP (U/L)	18.5 (12; 23)	21 (15; 30) *

Data given are means ± SD, or medians with interquartile ranges. ALT is defined as alanine aminotransferase, AST is defined as aspartate aminotransferase, GGTP is defined as gamma-glutamyl transpeptidase, MCV is defined as mean corpuscular volume, MCH is defined as mean hemoglobin content, MCHC is defined as mean corpuscular hemoglobin concentration, MPV is defined as mean platelet volume, PT is defined as prothrombin time, INR is defined as international normalized ratio, eGFR is defined as estimated glomerular filtration rate, CKD-EPI is defined as chronic kidney disease-epidemiological collaboration, CRP is defined as C-Reactive protein, RTH is defined as radiotherapy, and CHT is defined as chemotherapy. * *p* < 0.05, ** *p* < 0.01, *** *p* < 0.001.

## Data Availability

Not applicable.
